# Differential Level of RXFP3 Expression in Dopaminergic Neurons Within the Arcuate Nucleus, Dorsomedial Hypothalamus and Ventral Tegmental Area of RXFP3-Cre/tdTomato Mice

**DOI:** 10.3389/fnins.2020.594818

**Published:** 2021-01-06

**Authors:** Lara M. Voglsanger, Justin Read, Sarah S. Ch’ng, Cary Zhang, Izel M. Eraslan, Laura Gray, Leni R. Rivera, Lee D. Hamilton, Richard Williams, Andrew L. Gundlach, Craig M. Smith

**Affiliations:** ^1^Faculty of Health, School of Medicine, Institute for Innovation in Mental and Physical Health and Clinical Translation, Deakin University, Waurn Ponds, VIC, Australia; ^2^The Florey Institute of Neuroscience and Mental Health, The University of Melbourne, Parkville, VIC, Australia; ^3^Faculty of Health, School of Exercise and Nutritional Science, Deakin University, Waurn Ponds, VIC, Australia

**Keywords:** RXFP3, relaxin-3, dopamine, tyrosine hydroxylase, arcuate nucleus, dorsomedial hypothalamus

## Abstract

RXFP3 (relaxin-family peptide 3 receptor) is the cognate G-protein-coupled receptor for the neuropeptide, relaxin-3. RXFP3 is expressed widely throughout the brain, including the hypothalamus, where it has been shown to modulate feeding behavior and neuroendocrine activity in rodents. In order to better characterize its potential mechanisms of action, this study determined whether RXFP3 is expressed by dopaminergic neurons within the arcuate nucleus (ARC) and dorsomedial hypothalamus (DMH), in addition to the ventral tegmental area (VTA). Neurons that express RXFP3 were visualized in coronal brain sections from RXFP3-Cre/tdTomato mice, which express the tdTomato fluorophore within RXFP3-positive cells, and dopaminergic neurons in these areas were visualized by simultaneous immunohistochemical detection of tyrosine hydroxylase-immunoreactivity (TH-IR). Approximately 20% of ARC neurons containing TH-IR coexpressed tdTomato fluorescence, suggesting that RXFP3 can influence the dopamine pathway from the ARC to the pituitary gland that controls prolactin release. The ability of prolactin to reduce leptin sensitivity and increase food consumption therefore represents a potential mechanism by which RXFP3 activation influences feeding. A similar proportion of DMH neurons containing TH-IR expressed RXFP3-related tdTomato fluorescence, consistent with a possible RXFP3-mediated regulation of stress and neuroendocrine circuits. In contrast, RXFP3 was barely detected within the VTA. TdTomato signal was absent from the ARC and DMH in sections from Rosa26-tdTomato mice, suggesting that the cells identified in RXFP3-Cre/tdTomato mice expressed authentic RXFP3-related tdTomato fluorescence. Together, these findings identify potential hypothalamic mechanisms through which RXFP3 influences neuroendocrine control of metabolism, and further highlight the therapeutic potential of targeting RXFP3 in feeding-related disorders.

## Introduction

Relaxin-family peptide 3 receptor (RXFP3) is a G-protein-coupled receptor that is primarily expressed in the brain, and is activated by its cognate neuropeptide, relaxin-3 (Ma et al., 2017). Pharmacological manipulation of RXFP3 alters feeding behaviors in rodents, identifying RXFP3 as a promising therapeutic target for treating obesity and other eating disorders (McGowan et al., 2005, 2006; Ganella et al., 2012, 2013; Smith et al., 2014; De Ávila et al., 2018). For example, acute intracerebroventricular (ICV) administration of human relaxin-3 increased food consumption in rats (McGowan et al., 2005). As exogenous relaxin-3 pharmacologically cross-reacts with RXFP1, the preferred receptor for the hormone relaxin (Haugaard-Kedstrom et al., 2011), selective RXFP3 agonists and antagonists have been developed that provide more selective pharmacological modulation of RXFP3 (Ganella et al., 2012, 2013; Shabanpoor et al., 2012; Smith et al., 2014; De Ávila et al., 2018; Lee et al., 2020). These agonists also elicit orexigenic effects in rats and their actions are inhibited by co-injection of an RXFP3 antagonist (Haugaard-Kedstrom et al., 2011; Shabanpoor et al., 2012; De Ávila et al., 2018). Although RXFP3 agonists are not orexigenic in mice (Smith et al., 2013; Grosse et al., 2014), ICV administration of a selective RXFP3 antagonist has anorexigenic effects in this species, highlighting a role of endogenous relaxin-3/RXFP3 in feeding (Smith et al., 2014).

RXFP3 is broadly expressed throughout the mouse brain (Smith et al., 2010; Ma et al., 2017), including in hypothalamic nuclei such as the arcuate nucleus (ARC) and the dorsomedial hypothalamus (DMH) (Smith et al., 2010; Grosse et al., 2014). These brain regions are strongly implicated in feeding regulation (e.g., Abizaid et al., 2006; Marchant et al., 2010; Sohn, 2015), suggesting that RXFP3 within the hypothalamus plays a role in mediating the observed effects of relaxin-3 and RXFP3 agonists on feeding. Indeed, relaxin-3 microinjections within the ARC increased feeding in rats (McGowan et al., 2007). Similar effects were also observed following targeted injections into neighboring hypothalamic regions (McGowan et al., 2006; Ganella et al., 2012, 2013; De Ávila et al., 2018).

Indeed, the ARC is a major mediator of orexigenic and anorexigenic signaling within the brain (e.g., Abizaid et al., 2006; Sohn, 2015; Andermann and Lowell, 2017). The ARC is highly neurochemically heterogeneous and comprises multiple neuronal populations, which are characterized for research purposes as subpopulations that express characteristic markers such as cocaine-and amphetamine regulated transcript (CART), neuropeptide Y, agouti-related peptide (AgRP), and pro-opiomelanocortin (POMC) (Sohn, 2015; Andermann and Lowell, 2017; Campbell et al., 2017). In order to characterize how RXFP3 signaling in the hypothalamus can influence feeding, metabolism and related neuroendocrine events, it is crucial to identify which neuronal populations express RXFP3 within key areas such as the ARC. The ARC contains dopaminergic neurons (Brown et al., 2010) that are an essential component of the prolactin negative-feedback loop, and are involved in appetite regulation (Zhang and Van Den Pol, 2016). In addition to prolactin’s classical roles in lactation, it also decreases leptin sensitivity within the hypothalamus and thus increases food consumption in response to, and in preparation for, greater metabolic demands during the perinatal period (Grattan, 2015). In male rodents, prolactin has been shown to inhibit leptin release from adipose tissue (Brandebourg et al., 2007), suggesting that prolactin’s effect on leptin is not sex-specific. Dopaminergic neurons are also present within the neighboring DMH, but their specific physiological roles are not well characterized (Pirník et al., 2018).

Extrahypothalamic expression of RXFP3 in feeding-related brain areas has been documented, including in the ventral tegmental area (VTA) (Smith et al., 2010), which contains a major population of dopaminergic neurons that form the mesolimbic dopaminergic pathway. This pathway drives motivation and goal-directed behavior for both natural (e.g., food) and other (e.g., drug) rewards (Volkow et al., 2017).

The aim of this study was to determine whether RXFP3 is expressed by dopaminergic neurons within the ARC, DMH and VTA, to identify or exclude potential direct RXFP3-mediated effects on neurons in these areas. As a fully validated RXFP3 antibody is not yet available, transgenic RXFP3-Cre/tdTomato mice were used to visualize RXFP3-expressing neurons (Ch’ng et al., 2019). Histochemical detection of tdTomato fluorescence was combined with immunohistochemical detection of tyrosine hydroxylase (TH), an enzyme involved in the synthesis of catecholamines (Björklund and Dunnett, 2007), including dopamine (Daubner et al., 2011). TH is a highly reliable and commonly used marker for dopaminergic neurons, and the vast majority of neurons containing TH immunoreactivity (TH-IR) within the ARC, DMH, and VTA have been confirmed as dopaminergic (Zhang et al., 2010; Zhang and Van Den Pol, 2015).

## Materials and Methods

### RXFP3-Cre/tdTomato and Rosa26-tdTomato Mice

All experiments were performed in accordance with the Prevention of Cruelty to Animals Act 1986 under guidelines of the National Health and Medical Research Council Code of Practice for the Care and Use of Animals for Experimental Purposes in Australia. These experiments were approved by the Florey Institute of Neuroscience and Mental Health Animal Ethics Committee (#14-035-FINMH).

RXFP3-Cre/tdTomato double transgenic mice (heterozygous for each mutation) were bred by pairing separate homozygous RXFP3-Cre and Rosa26-tdTomato reporter strains, as described (Ch’ng et al., 2019). The RXFP3-Cre [Tg(Rxfp3-cre), RS38Gsat] strain was maintained on a mixed background of FVB/N and Crl:CD1 (ICR; obtained from MMRRC/Gensat, stock no. 036667-UCD). In these mice, a Cre-expression cassette was inserted at the initiating ATG codon of the first coding exon of the *Rxfp3* gene on BAC clone RP23-220A13, allowing the additional BAC *Rxfp3* gene to express Cre-recombinase. Random insertion of this BAC therefore confers Cre-recombinase expression only within RXFP3-expressing (RXFP3+) cells. The Rosa26-tdTomato line [Ai14(RCL-tdT)-D; Jackson Laboratory, stock no. 007914, RRID:IMSR_JAX:007914] contains a floxed stop codon upstream of the tdTomato coding region inserted at the Rosa26 locus (Madisen et al., 2010), enabling Cre-dependent expression of this fluorophore solely within RXFP3+ cells in double transgenic RXFP3-Cre/tdTomato mice.

### Tissue Collection and Preparation

Male 8 week-old RXFP3-Cre/tdTomato mice (*n* = 5) and an 11 week-old Rosa26-tdTomato mouse (*n* = 1) were anaesthetized with an intraperitoneal injection of sodium pentobarbitone (80 mg/kg, 0.1 mL/10 g body weight). Using phosphate-buffered saline (PBS, 0.1 M, pH 7.4), mice were transcardially perfused for 90 s at a flow rate of 7 mL/min, followed by 4% paraformaldehyde (PFA) for 6 min in PBS. Brains were removed and post-fixed at 4°C in 4% PFA in PBS for 1 h, followed by cryoprotection in 20% sucrose in PBS at 4°C overnight. Brains were then frozen over dry ice and stored at –80°C until sectioning. Brains were mounted in a cryostat using Tissue-Plus Optimal Cutting Temperature medium (Cat#23-730-625, SciGen, Belrose, NSW, Australia), and coronal sections (40 μm) from RXFP3-Cre/tdTomato mice were placed in 12-well plates containing cryoprotectant [33% ethylene glycol (Cat#EA007-500M, Chem Supply, Gillman, SA, Australia), 33% glycerol (Cat#242-500ml, Univar, Singapore) in PBS] and stored at –20°C until further use. Sections from the Rosa26-tdTomato mouse were instead collected into PBS, mounted onto glass slides and coverslipped as below.

### Immunohistochemistry

Brain sections from RXFP3-Cre/tdTomato mice were removed from cryoprotectant and placed into mesh inserts (to reduce handling damage) in a 6-well tissue culture plate containing phosphate buffer (PB; 2.7 mM KCl, 11.2 mM Na_2_HPO_4_, 1.8 mM KH_2_PO_4_, pH 7.4). Sections were washed in PB containing 0.05% Triton-X for 4 × 5 min on an orbital mixer, followed by 4 × 5 min PB. Sections were then incubated in blocking solution at room temperature [10% normal donkey serum (NDS; Cat#S30-100ML, Merck, Castle Hill, NSW, Australia) in PB] for a minimum of 1 h, and left overnight in PB with 2% NDS and rabbit anti-TH primary antibody at room temperature (1:1,000 dilution; Cat#AB152, Millipore, Bayswater, VIC, Australia). The next day, sections were washed in PB for 6 × 5 min, and incubated for a minimum of 1 h in PB with 2% NDS and donkey anti-rabbit Alexa488 secondary antibody (1:500 dilution; Cat#ab150061, Abcam, Melbourne, VIC, Australia). Sections were washed in PB for 5 × 5 min, mounted on glass microscope slides and coverslipped using DAKO fluorescent mounting medium (Cat#S302380-2, Agilent, Mulgrave, VIC, Australia) before storage at 4°C.

### Microscopy, Data Collection, and Statistical Analysis

A single, representative image from each of three (3) RXFP3-Cre/tdTomato mice through each area of interest was obtained using a 40 × objective on a Leica Confocal SP5 microscope (Leica, Macquire Park Germany). Using the Allen Institute Mouse Brain Reference Atlas (Lein et al., 2007) to identify surrounding visual markers, images of coronal brain sections were obtained specifically focusing on the ARC, DMH and VTA (–2.06, –2.06, and –3.6 mm A/P from bregma, respectively). Manual cell quantification was undertaken using ImageJ software, whereby individual neurons in separate channels were counted/tagged, before images were merged to determine co-localisation counts. Graphs were generated and data were analyzed using Graphpad Prism software (V8; Graphpad, San Diego, CA, United States), and statistical comparisons between groups were performed via parametric paired *t*-tests through *p* value (p), *t* ratio (t), and correlation coefficient (r). A single representative image through the ARC and DMH from a Rosa26-tdTomato mouse was collected as above, which revealed an absence of strong tdTomato-expressing cells within these regions.

## Results

**FIGURE 1:**
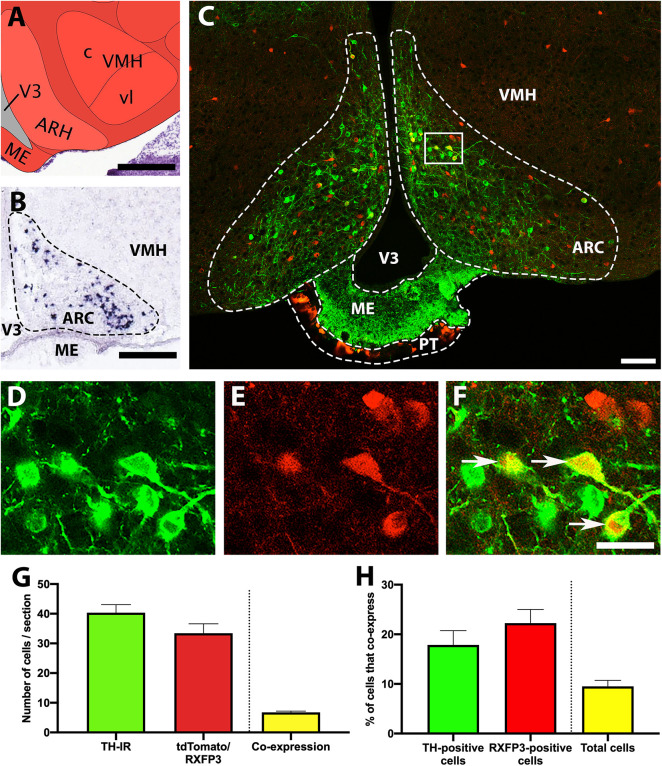
Co-localisation of TH-IR and tdTomato in the ARC of RXFP3-Cre/tdTomato mice. **(A)** Schematic image of the coronal level of the ARC analyzed, from the Allen Institute Reference Brain Atlas (Plate 75; http://atlas.brain-map.org/). **(B)** Allen Brain Atlas *in situ* hybridisation image, illustrating TH mRNA within the ARC (Plate 22). **(C)** Confocal image of a representative coronal section through the ARC in RXFP3-Cre/tdTomato mice, illustrating tdTomato-expressing neurons (red) and TH-IR neurons (green). **(D)** Magnified image of area surrounded by rectangle in **(C)**, illustrating TH-IR cells (green), **(E)** tdTomato-expressing neurons (red), and **(F)** combined. Double-labeled neurons (yellow) are indicated by arrows. **(G)** Number of cells within the ARC per section (from *n* = 3 mice) that displayed TH-IR, tdTomato expression, and both (double-labeled). **(H)** Percentage of TH-IR cells within the ARC that co-expressed tdTomato, the percentage of tdTomato-expressing cells that co-expressed TH-IR, and the percentage of the total number of cells counted (sum of tdTomato + TH-IR) that displayed co-expression. Data are expressed as mean ± SEM. Scale bars, **(A)** 130 μm, **(B)** 100 μm, **(C)** 50 μm, **(F)** 20 μm. V3, 3rd ventricle; ARH/ARC, arcuate nucleus; ME, median eminence; PT, pars tuberalis of the pituitary gland; VMH, ventromedial hypothalamic nucleus.

*In situ* hybridisation data from the Allen Brain Atlas (Lein et al., 2007) demonstrates that TH mRNA is highly expressed within the ARC (Figures 1A,B), and a similar distribution of neurons containing TH-IR was observed in RXFP3-Cre/tdTomato mice (Figure 1C). TdTomato fluorescence was equally pronounced within the ARC (but not adjacent regions), and neurons that co-expressed both markers were easily identified (Figures 1D–F). TH-IR- and tdTomato-positive cells were present in roughly equal numbers (between 35 and 40 cells/section; *p* = 0.271, *t* = 1.5, *r* = –0.19; Figure 1G). Approximately 20% of these two neuronal populations expressed both markers (*p* = 0.146, *t* = 2.3, *r* = 0.77; Figure 1H), and when the total number of both tdTomato and TH-IR cells were summed, approximately 10% exhibited double-labeling. A high density of tdTomato-positive cells (and neuronal fibers) was also observed surrounding the median eminence in the *pars tuberalis* of the pituitary gland (PT; Figure 1C; data not quantified).

**FIGURE 2:**
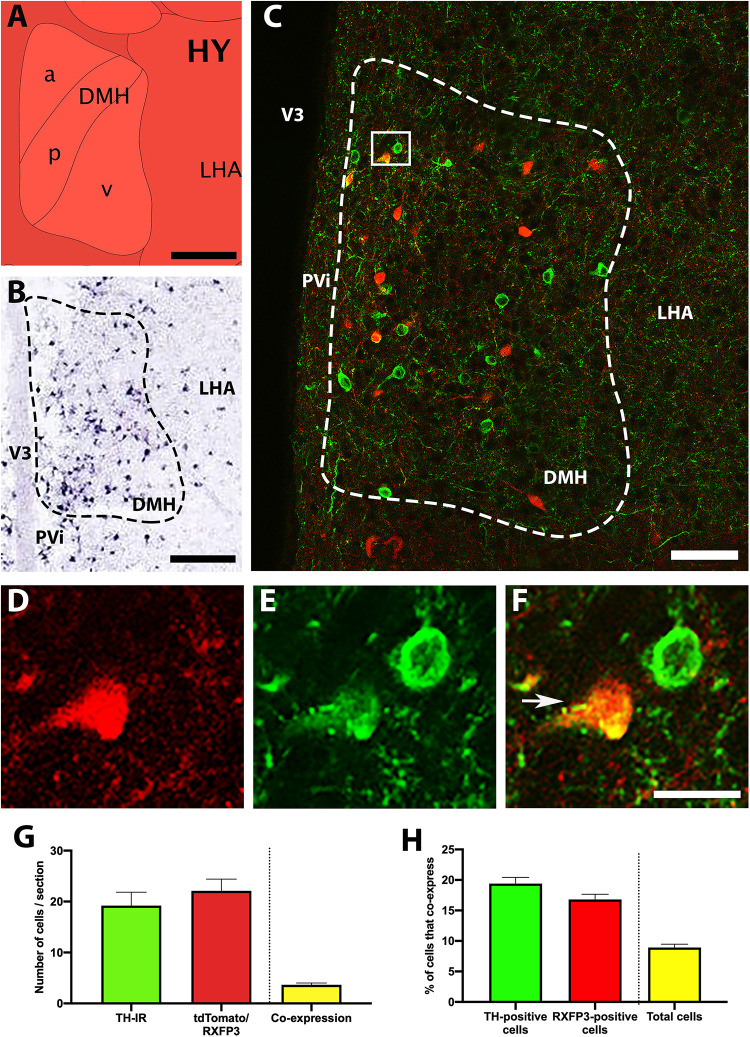
Co-localisation of TH-IR and tdTomato in the DMH of RXFP3-Cre/tdTomato mice. **(A)** Schematic image of the coronal level of the DMH analyzed from the Allen Brain Atlas Reference Atlas (Plane 75; http://atlas.brain-map.org/). **(B)** Allen Brain Atlas *in situ* hybridisation image, demonstrating TH mRNA within the DMH (Plate 22). **(C)** Confocal image of a representative coronal section through the DMH in RXFP3-Cre/tdTomato mice, illustrating tdTomato-expressing neurons (red) and TH-IR neurons (green). **(D)** Magnified image of area surrounded by rectangle in **(C)**, illustrating TH-IR cells (green), **(E)** tdTomato-expressing cells (red), and **(F)** combined, where a co-expressing cell (yellow) is indicated by an arrow. **(G)** Number of cells within the DMH per section (from *n* = 3 mice) that displayed TH-IR, tdTomato expression, and both (double labeled). **(H)** Percentage of TH-IR cells within the DMH that co-expressed tdTomato, the percentage of tdTomato-expressing cells that co-expressed TH-IR, and the percentage of the total number of cells counted (sum of tdTomato + TH-IR) that displayed co-expression. Data are expressed as mean ± SEM. Scale bars, **(A)** 130 μm, **(B)** 100 μm, **(C)** 50 μm, **(F)** 20 μm. PVi, periventricular hypothalamic nucleus; DMH, dorsomedial hypothalamus; LHA, lateral hypothalamus.

TH mRNA is highly expressed within the DMH (Figures 2A,B; Lein et al., 2007), and neurons containing TH-IR were clearly visible within this region, but not surrounding regions (Figure 2C). Similar to the ARC, roughly equal numbers of cells containing TH-IR and tdTomato-fluorescence were present in the DMH (between 19 and 23 cells/section; *p* = 0.122, *t* = 2.6, *r* = 0.91; Figures 2D–G). Approximately 20% of both TH-IR- and tdTomato-positive neurons were double-labeled (*p* = 0.183, *t* = 2.0, *r* = 0.05; Figure 2H), which equated to less than 10% of the total number of cells positive for either signal displaying coexpression.

**FIGURE 3:**
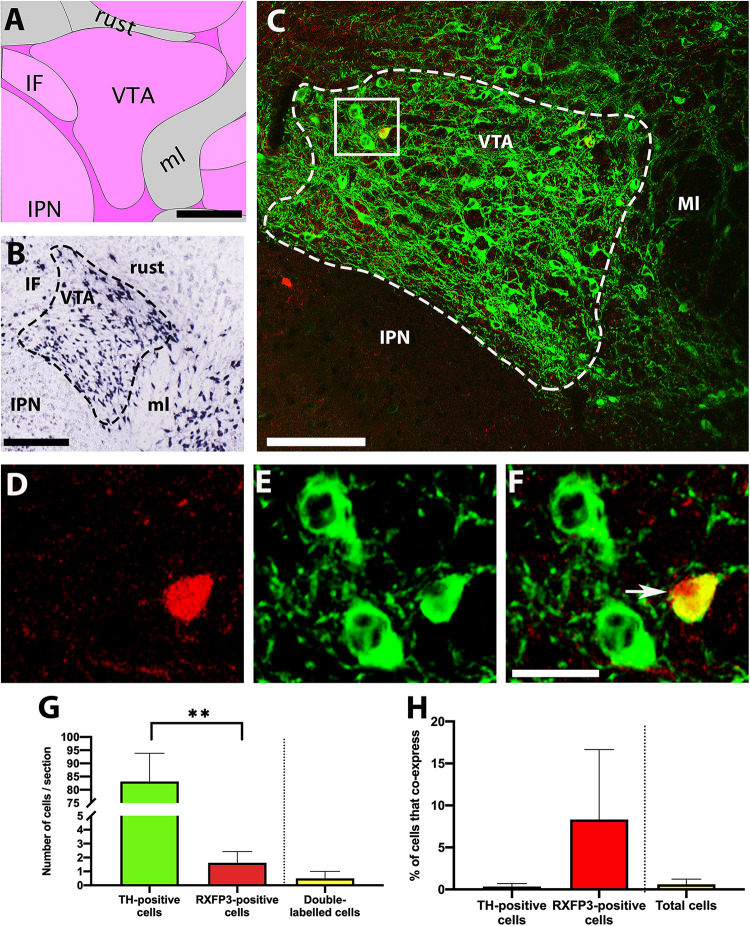
Co-localisation of TH-IR and tdTomato in the VTA of RXFP3-Cre/tdTomato mice. **(A)** Schematic image of the coronal level of the VTA analyzed from the Allen Institute Reference Brain Atlas (Plate 87; http://atlas.brain-map.org/). **(B)** Allen Brain Atlas *in situ* hybridisation image, demonstrating TH mRNA within the VTA (Plate 30). **(C)** Confocal image of a representative coronal section through the VTA in RXFP3-Cre/tdTomato mice, illustrating tdTomato-expressing neurons (red) and TH-IR neurons (green). **(D)** Magnified image of area surrounded by rectangle in **(C)**, illustrating TH-IR cells (green), **(E)** a tdTomato-expressing cell (red), and **(F)** combined. Double-labeled cell (yellow) is indicated by an arrow. **(G)** Number of cells within the VTA per section (from *n* = 3 mice) that displayed TH-IR, tdTomato expression, and both (co-expression) ^∗∗^*p* < 0.01. **(H)** Percentage of TH-IR cells within the VTA that co-expressed tdTomato, the percentage of tdTomato-expressing cells that co-expressed TH-IR, and the percentage of the total number of cells counted (sum of tdTomato + TH-IR) that displayed co-expression. Data expressed as mean ± SEM. Scale bars, **(A)** 200 μm, **(B)** 270 μm, **(C)** 50 μm, **(F)** 20 μm. IF, interfascicular nucleus raphe; rust, rubrospinal tract; ml, medial lemniscus; IPN, interpeduncular nucleus; VTA, ventral tegmental area.

Similarly, *in situ* hybridisation data from the Allen Brain Atlas reveals high levels of TH mRNA within the VTA (Figures 3A,B; Lein et al., 2007). Although the two-dimensional area occupied by the VTA in coronal sections changes substantially in the anterior-posterior direction, its borders were clearly delineated in our sections by the distribution of neurons containing TH-IR. This aided the identification of VTA-containing sections at a consistent coronal level in different RXFP3-Cre/tdTomato mice, at which TH-IR neurons were similarly enriched (Figure 3C). Very low numbers of tdTomato-positive cells were identified in this region (Figures 3D–F), in contrast to the large number of TH-IR neurons observed (*p* = 0.004, *t* = 8.1, *r* = 0.74; Figure 3G). Sparse double-labeled cells were identified in some of the sections analyzed, but this represented essentially 0% cells containing TH-IR co-labeled, and a small percentage of tdTomato-positive cells expressed TH-IR (*p* = 0.391, *t* = 1.0, *r* = 1.0; Figure 3H). There was also essentially 0% of the total number of cells positive for either signal that displayed coexpression.

**FIGURE 4:**
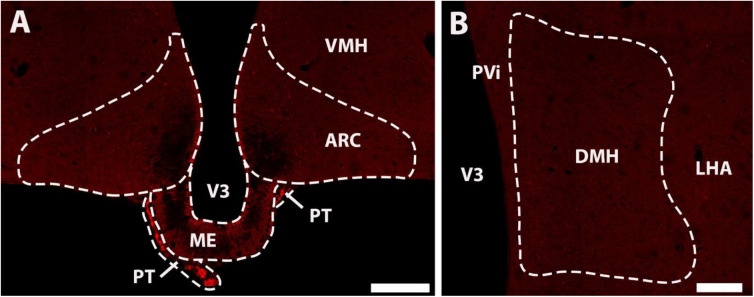
Confocal images illustrating a lack of tdTomato-expressing neurons in representative coronal sections from a Rosa26-tdTomato mouse, through the ARC **(A)**, and DMH **(B)**. Notably, tdTomato-expressing cells were observed within the PT **(A)**, indicating that this expression is unrelated to RXFP3 expression. Scale bars, 20 μm. ARC, arcuate nucleus; DMH, dorsomedial hypothalamus; LHA, lateral hypothalamus; ME, median eminence; PT, pars tuberalis of the pituitary gland; PVi, periventricular hypothalamic nucleus; V3, 3rd ventricle; VMH, ventromedial hypothalamic nucleus.

In order to confirm that tdTomato expression within the ARC and DMH of double mutant RXFP3-Cre/tdTomato mice is Cre-dependent, a single mutant Rosa26-tdTomato mouse was analyzed. Bright tdTomato-expressing cells were absent within the ARC (Figure 4A) and DMH (Figure 4B) of these mice. Interestingly however, a high density of bright tdTomato-fluorescence was present within the PT (Figure 4A), which demonstrates that tdTomato-expression within this area does not indicate the presence of Cre or RXFP3 in RXFP3-Cre/tdTomato mice.

## Discussion

A key finding of this study was that a small population of dopaminergic neurons within the ARC express RXFP3. It is therefore feasible, that RXFP3 activation inhibits this small proportion of ARC dopaminergic neurons, as electrophysiological studies suggest that postsynaptic RXFP3 signaling is predominantly inhibitory in rat and mouse (Kania et al., 2017; Ch’ng et al., 2019; Kania et al., 2020). These data are in line with cell-based experiments demonstrating that RXFP3 activation reduces intracellular cAMP (see Kocan et al., 2014 for review). Although the functional significance of this modest expression of RXFP3 by ARC dopaminergic neurons is currently unknown, it is possible that modulation of this population could contribute to the effects of centrally administered RXFP3 agonists on feeding and neuroendocrine function.

Prolactin binds to prolactin receptors on ARC dopaminergic neurons (Lerant and Freeman, 1998), which normally stimulates the release of dopamine into the portal blood system causing negative feedback and reduced prolactin release. Potential RXFP3-mediated inhibition of ARC dopaminergic neurons may therefore cause more subtle/reduced dopamine release into the portal blood system, resulting in greater activation of lactotroph cells in the anterior pituitary (Mansour et al., 1990) and increased prolactin secretion. Prolactin increases feeding by decreasing leptin sensitivity, and this process involves increased expression of hypothalamic suppressor of cytokine signaling (SOCS) proteins (Buonfiglio et al., 2016). In a powerful demonstration of the cross-talk between leptin and prolactin, chronic ICV injections of prolactin countered the anorexigenic effects of acute leptin administration (Grattan, 2015; Buonfiglio et al., 2016). Interestingly, increased plasma leptin has been observed following both acute (McGowan et al., 2014) and chronic (Hida et al., 2006; McGowan et al., 2006) injection of relaxin-3 into rat brain. Further investigating the possible influence of RXFP3 on leptin pathways is therefore of interest, especially as the actions of RXFP3 likely involve changes in gene expression and other medium to long-term effects, as well as rapid neuronal inhibition (or activation) (McGowan et al., 2006; Kocan et al., 2014).

Notably however, a majority of the ARC tdTomato (RXFP3)-positive cells were negative for TH-IR. In light of the neurochemical heterogeneity of this region, with several populations characterized by their expression of CART, neuropeptide Y and other peptidergic markers (Campbell et al., 2017), TH-negative/RXFP3-positive neurons in the ARC may constitute one or more of these other populations. ARC CART neurons express leptin receptors (Kristensen et al., 1998), and trigger anorexigenic pathways and reduced food consumption (Ahmadian-Moghadam et al., 2018). It is therefore possible that RXFP3 may be expressed by ARC CART neurons, and that RXFP3-mediated inhibition of these neurons may contribute to the orexigenic actions of RXFP3. Also of note, the number and density of tdTomato-positive cells within the ARC was greater than reported in our previous study (Smith et al., 2010), which detected *Rxfp3* mRNA using radioactive *in situ* hybridisation histochemistry. This discrepancy may be due to an increased sensitivity of target detection provided by the transgenic RXFP3-Cre/tdTomato mice, whereby cells that express *Rxfp3* mRNA at low levels, still express sufficient Cre-recombinase to drive robust expression of tdTomato, which is driven by a strong promoter at the Rosa26 locus. The different strains of mice used in these studies may also contribute to this differential observation.

Another key finding of this study was that within the DMH, a relatively low level of coexpression of TH-IR and tdTomato fluorescence was observed. The functional relevance of this co-expression is currently unknown, especially since the role of DMH dopaminergic neurons remains largely unexplored. However, the DMH is considered to be a relay station for multiple feeding, neuroendocrine and stress pathways, as it receives multiple inputs from the majority of hypothalamic regions including the ARC (Pirník et al., 2018), and has complex outputs to autonomic and other pathways (Brasil et al., 2020). Notably, it contains a range of anorexigenic neuron populations that may express RXFP3, including cholinergic and proopiomelanocortin (POMC) neurons. Furthermore, prolactin-releasing peptide is also produced by a subset of DMH neurons (Jeong et al., 2017; Pirník et al., 2018). Lesions of the DMH result in decreased food intake, but the precise contributions of different neuronal subpopulations in this effect requires further investigation (Jeong et al., 2017).

In this study, tdTomato was not consistently observed in VTA dopaminergic neurons, suggesting that RXFP3 is unlikely to have a direct influence on VTA neuron activity. A previous study reported a high level of RXFP3 in the VTA using immunohistochemistry with a non-validated, (and commercially discontinued) anti-RXFP3 antibody (Paul et al., 2019). Antibodies that have high sensitivity and specificity against G-protein coupled receptors (GPCRs) such as RXFP3 are rare, as receptor antigens usually misfold once they are no longer in the lipid bilayer following injection into animals (Michel et al., 2009). Therefore, this may explain the discrepancy between this previous immunohistochemical study, and the finding of low VTA tdTomato described here. However, earlier *in situ* hybridisation studies also suggest moderate levels of *Rxfp3* mRNA are present within the VTA (Lein et al., 2007; Smith et al., 2010). Paul and colleagues also observed that *Rxfp3* mRNA was enriched within GABAergic VTA neurons, and that relatively more RXFP3-expressing neurons were present within the rostromedial regions of this structure (Paul et al., 2019), which is slightly different to the more caudal/central coronal level studied here. Further studies using other methods to detect RXFP3 in this area are therefore warranted, as transgenic mouse strains such as the one used here are sometimes prone to false-negative detection of markers (see below).

Lastly, the absence of bright tdTomato-expressing cells within the ARC and DMH of a Rosa26-tdTomato mouse demonstrated that tdTomato expression is Cre-dependent (and therefore, represents RXFP3) within these areas in RXFP3-Cre/tdTomato mice. However, the presence of bright tdTomato-expressing cells within the PT of a Rosa26-tdTomato mouse suggests that RXFP3 is absent from this structure, and highlights the importance of examining these mice as controls (Madisen et al., 2010). Further studies to investigate the potential expression and function of RXFP3 within other regions of the pituitary gland, are nonetheless warranted. For example, RXFP3 expression was recently detected in the LβT2 mouse pituitary gonadotroph cell line (Kanasaki et al., 2019), and data from the Genotype-Tissue Expression (GTEx) project describes RXFP3 expression within human pituitary (and adrenal) samples^1^. The potential source of relaxin-3 that might access these receptors is unknown, however. Furthermore, several rodent studies have observed that relaxin-3 can influence the hypothalamic-pituitary adrenal and gonadal axes, although some of these effects may be due to relaxin-3 binding to RXFP1, rather than RXFP3 (Zhang et al., 2017; De Ávila et al., 2018).

Investigating the identity and function of RXFP3-positive cells in other brain areas may also provide mechanistic insights into how RXFP3 modulates feeding behavior and relevant neuroendocrine systems. In this regard, a previous study observed direct RXFP3-mediated inhibition of oxytocin and vasopressin neurons within the rat paraventricular hypothalamic nucleus (PVN) (Kania et al., 2017). A recent follow-up study characterized these mechanisms further, and demonstrated that local PVN injection of an RXFP3 antagonist prevented binge eating in female rats (Kania et al., 2020). The absence of tdTomato fluorescence within the PVN of RXFP3-Cre/tdTomato mice was therefore notable, particularly as previous *in situ* hybridisation studies have demonstrated high levels of *Rxfp3* mRNA expression within this structure (Smith et al., 2010; Ma et al., 2017). The reason for this discrepancy is unknown, but may be due to the different mouse strains used or may represent false-negative fluorophore expression, whereby the fluorophore fails to be faithfully expressed within some neuronal populations, an issue often associated with transgenic reporter mice (Yang and Gong, 2005).

In summary, this study identified that a small proportion of hypothalamic dopaminergic neurons express RXFP3. Of particular interest, the detection of a small population of ARC dopaminergic neurons that express RXFP3 highlights a novel potential mechanism by which RXFP3 activation may inhibit these neurons to promote food intake and influence neuroendocrine functions.

## Data Availability Statement

The raw data supporting the conclusions of this article will be made available by the authors, without undue reservation, to any qualified researcher.

## Ethics Statement

The animal study was reviewed and approved by the Florey Institute of Neuroscience and Mental Health Animal Ethics Committee.

## Author Contributions

AG and CS conceived the project and designed the study. LV performed the experiments and completed the analysis, including preparation of the figures, with contributions from SC, CZ, and IE. LV and CS wrote the manuscript. JR, LG, LR, LH, and RW provided technical advice, project guidance, and other assistance. All authors provided editorial input on, and approval of, the final manuscript.

## Conflict of Interest

The authors declare that the research was conducted in the absence of any commercial or financial relationships that could be construed as a potential conflict of interest.

## Footnotes

1 www.gtexportal.org/home/gene/RXFP3

## References

[B1] Abizaid A. Gao Q. Horvath T. L. (2006). Thoughts for food: brain mechanisms and peripheral energy balance. *Neuron* 51 691–702. 10.1016/j.neuron.2006.08.025 16982416

[B2] Ahmadian-Moghadam H. Sadat-Shirazi M.-S. Zarrindast M.-R. (2018). Cocaine-and amphetamine-regulated transcript (CART): a multifaceted neuropeptide. *Peptides* 110 56–77. 10.1016/j.peptides.2018.10.008 30391426

[B3] Andermann M. L. Lowell B. B. (2017). Toward a wiring diagram understanding of appetite control. *Neuron* 95 757–778. 10.1016/j.neuron.2017.06.014 28817798 PMC5657399

[B4] Björklund A. Dunnett S. B. (2007). Dopamine neuron systems in the brain: an update. *Trends Neurosci.* 30 194–202. 10.1016/j.tins.2007.03.006 17408759

[B5] Brandebourg T. D. Bown J. L. Ben-Jonathan N. (2007). Prolactin upregulates its receptors and inhibits lipolysis and leptin release in male rat adipose tissue. *Biochem. Biophys. Res. Commun.* 357 408–413. 10.1016/j.bbrc.2007.03.168 17433256 PMC1885988

[B6] Brasil T. F. S. Lopes-Azevedo S. Belém-Filho I. J. A. Fortaleza E. A. T. Antunes-Rodrigues J. Corrêa F. M. A. (2020). The dorsomedial hypothalamus is involved in the mediation of autonomic and neuroendocrine responses to restraint stress. *Front. Pharmacol.* 10:1547 10.3389/fphar.2019.01547 PMC6989482 32038236

[B7] Brown R. S. E. Kokay I. C. Herbison A. E. Grattan D. R. (2010). Distribution of prolactin-responsive neurons in the mouse forebrain. *J. Comp. Neurol.* 518 92–102. 10.1002/cne.22208 19882722

[B8] Buonfiglio D. C. Ramos-Lobo A. M. Freitas V. M. Zampieri T. T. Nagaishi V. S. Magalhães M. (2016). Obesity impairs lactation performance in mice by inducing prolactin resistance. *Sci. Rep.* 6:22421. 10.1038/srep22421 PMC4772384 26926925

[B9] Campbell J. N. Macosko E. Z. Fenselau H. Pers T. H. Lyubetskaya A. Tenen D. (2017). A molecular census of arcuate hypothalamus and median eminence cell types. *Nat. Neurosci.* 20 484–496. 10.1038/nn.4495 28166221 PMC5323293

[B10] Ch’ng S. S. Fu J. Brown R. M. Smith C. M. Hossain M. A. McDougall S. J. (2019). Characterization of the relaxin family peptide receptor 3 system in the mouse bed nucleus of the stria terminalis. *J. Comp. Neurol.* 527 2615–2633. 10.1002/cne.24695 30947365

[B11] Daubner S. C. Le T. Wang S. (2011). Tyrosine hydroxylase and regulation of dopamine synthesis. *Arch. Biochem. Biophys.* 508 1–12. 10.1016/j.abb.2010.12.017 21176768 PMC3065393

[B12] De Ávila C. Chometton S. Lenglos C. Calvez J. Gundlach A. L. Timofeeva E. (2018). Differential effects of relaxin-3 and a selective relaxin-3 receptor agonist on food and water intake and hypothalamic neuronal activity in rats. *Behav. Brain Res.* 336 135–144. 10.1016/j.bbr.2017.08.044 28864207

[B13] Ganella D. E. Callander G. E. Ma S. Bye C. R. Gundlach A. L. Bathgate R. A. (2013). Modulation of feeding by chronic rAAV expression of a relaxin-3 peptide agonist in rat hypothalamus. *Gene Ther.* 20 703–716. 10.1038/gt.2012.83 23135160

[B14] Ganella D. E. Ryan P. J. Bathgate R. A. Gundlach A. L. (2012). Increased feeding and body weight gain in rats after acute and chronic activation of RXFP3 by relaxin-3 and receptor-selective peptides: functional and therapeutic implications. *Behav. Pharmacol.* 23 516–525. 10.1097/fbp.0b013e3283576999 22854307

[B15] Grattan D. R. (2015). 60 Years of Neuroendocrinology: the hypothalamo-prolactin axis. *J. Endocrinol.* 226 T101–T122. 26101377 10.1530/JOE-15-0213 PMC4515538

[B16] Grosse J. Heffron H. Burling K. Hossain M. A. Habib A. M. Rogers G. J. (2014). Insulin-like peptide 5 is an orexigenic gastrointestinal hormone. *Proc. Natl. Acad. Sci. (USA)* 111 11133–11138. 25028498 10.1073/pnas.1411413111 PMC4121845

[B17] Haugaard-Kedstrom L. M. Shabanpoor F. Hossain M. A. Clark R. J. Ryan P. J. Craik D. J. (2011). Design, synthesis, and characterization of a single-chain peptide antagonist for the relaxin-3 receptor RXFP3. *J. Am. Chem. Soc.* 133 4965–4974. 10.1021/ja110567j 21384867

[B18] Hida T. Takahashi E. Shikata K. Hirohashi T. Sawai T. Seiki T. (2006). Chronic intracerebroventricular administration of relaxin-3 increases body weight in rats. *J. Recept. Signal. Transduct. Res.* 26 147–158. 10.1080/10799890600623373 16777712

[B19] Jeong J. H. Lee D. K. Jo Y.-H. (2017). Cholinergic neurons in the dorsomedial hypothalamus regulate food intake. *Mol. Metab.* 6 306–312. 10.1016/j.molmet.2017.01.001 28271037 PMC5323886

[B20] Kanasaki H. Tumurbaatar T. Tumurgan Z. Oride A. Okada H. Kyo S. (2019). Effect of relaxin-3 on Kiss-1, gonadotropin-releasing hormone, and gonadotropin subunit gene expression. *Reprod. Med. Biol.* 18 397–404. 10.1002/rmb2.12298 31607801 PMC6780024

[B21] Kania A. Gugula A. Grabowiecka A. De Ávila C. Blasiak T. Rajfur Z. (2017). Inhibition of oxytocin and vasopressin neuron activity in rat hypothalamic paraventricular nucleus by relaxin-3–RXFP3 signalling. *J. Physiol.* 595 3425–3447. 10.1113/jp273787 28098344 PMC5451722

[B22] Kania A. Szlaga A. Sambak P. Gugula A. Blasiak E. Di Bonaventura M. V. M. (2020). RLN3/RXFP3 signaling in the PVN inhibits magnocellular neurons via M-like current activation and contributes to binge eating behavior. *J. Neurosci.* 40 5362–5375. 10.1523/jneurosci.2895-19.2020 32532885 PMC7343322

[B23] Kocan M. Sarwar M. Hossain M. A. Wade J. D. Summers R. J. (2014). Signalling profiles of H3 relaxin, H2 relaxin and R3(BΔ23–27)R/I5 acting at the relaxin family peptide receptor 3 (RXFP3). *Br. J. Pharmacol.* 171 2827–2841. 10.1111/bph.12623 24641548 PMC4243858

[B24] Kristensen P. Judge M. E. Thim L. Ribel U. Christjansen K. N. Wulff B. S. (1998). Hypothalamic CART is a new anorectic peptide regulated by leptin. *Nature* 393 72–76. 10.1038/29993 9590691

[B25] Lee H. S. Postan M. Song A. Clark R. J. Bathgate R. A. Haugaard-Kedström L. M. (2020). Development of relaxin-3 agonists and antagonists based on grafted disulfide-stabilized scaffolds. *Front. Chem.* 8:87. 10.3389/fchem.2020.00087 32133341 PMC7039932

[B26] Lein E. S. Hawrylycz M. J. Ao N. Ayres M. Bensinger A. Bernard A. (2007). Genome-wide atlas of gene expression in the adult mouse brain. *Nature* 445 168–176. 17151600 10.1038/nature05453

[B27] Lerant A. Freeman M. E. (1998). Ovarian steroids differentially regulate the expression of PRL-R in neuroendocrine dopaminergic neuron populations: a double label confocal microscopic study. *Brain Res.* 802 141–154. 10.1016/s0006-8993(98)00583-6 9748546

[B28] Ma S. Smith C. M. Blasiak A. Gundlach A. L. (2017). Distribution, physiology and pharmacology of relaxin-3/RXFP3 systems in brain. *Br. J. Pharmacol.* 174 1034–1048. 10.1111/bph.13659 27774604 PMC5406293

[B29] Madisen L. Zwingman T. A. Sunkin S. M. Oh S. W. Zariwala H. A. Gu H. (2010). A robust and high-throughput Cre reporting and characterization system for the whole mouse brain. *Nat. Neurosci.* 13 133–140. 10.1038/nn.2467 20023653 PMC2840225

[B30] Mansour A. Meador-Woodruff J. Bunzow J. Civelli O. Akil H. Watson S. (1990). Localization of dopamine D2 receptor mRNA and D1 and D2 receptor binding in the rat brain and pituitary: an in situ hybridization-receptor autoradiographic analysis. *J. Neurosci.* 10 2587–2600. 10.1523/jneurosci.10-08-02587.1990 2143777 PMC6570265

[B31] Marchant N. J. Furlong T. M. McNally G. P. (2010). Medial dorsal hypothalamus mediates the inhibition of reward seeking after extinction. *J. Neurosci.* 30 14102–14115. 10.1523/jneurosci.4079-10.2010 20962231 PMC6634760

[B32] McGowan B. M. Minnion J. S. Murphy K. G. Roy D. Stanley S. A. Dhillo W. S. (2014). Relaxin-3 stimulates the neuro-endocrine stress axis via corticotrophin-releasing hormone. *J. Endocrinol.* 221 337–346. 10.1530/JOE-13-0603 24578294

[B33] McGowan B. M. Stanley S. A. Smith K. L. Minnion J. S. Donovan J. Thompson E. L. (2006). Effects of acute and chronic relaxin-3 on food intake and energy expenditure in rats. *Reg. Pept.* 136 72–77. 10.1016/j.regpep.2006.04.009 16764952

[B34] McGowan B. M. Stanley S. A. Smith K. L. White N. E. Connolly M. M. Thompson E. L. (2005). Central relaxin-3 administration causes hyperphagia in male Wistar rats. *Endocrinology* 146 3295–3300. 10.1210/en.2004-1532 15845619

[B35] McGowan B. M. Stanley S. A. White N. E. Spangeus A. Patterson M. Thompson E. L. (2007). Hypothalamic mapping of orexigenic action and Fos-like immunoreactivity following relaxin-3 administration in male Wistar rats. *Am. J. Physiol. Endocrinol. Metab.* 292 E913–E919. 17132825 10.1152/ajpendo.00346.2006

[B36] Michel M. C. Wieland T. Tsujimoto G. (2009). How reliable are G-protein-coupled receptor antibodies? *Naunyn Schmiedebergs Arch. Pharmacol.* 379 385–388. 10.1007/s00210-009-0395-y 19172248

[B37] Paul E. J. Tossell K. Ungless M. A. (2019). Transcriptional profiling aligned with in situ expression image analysis reveals mosaically expressed molecular markers for GABA neuron sub-groups in the ventral tegmental area. *Eur. J. Neurosci.* 50 3732–3749. 10.1111/ejn.14534 31374129 PMC6972656

[B38] Pirník Z. Kolesárová M. Železná B. Maletínská L. (2018). Repeated peripheral administration of lipidized prolactin-releasing peptide analog induces c-fos and FosB expression in neurons of dorsomedial hypothalamic nucleus in male C57 mice. *Neurochem. Int.* 116 77–84. 10.1016/j.neuint.2018.03.013 29601847

[B39] Shabanpoor F. Akhter Hossain M. Ryan P. J. Belgi A. Layfield S. Kocan M. (2012). Minimization of human relaxin-3 leading to high-affinity analogues with increased selectivity for relaxin-family peptide 3 receptor (RXFP3) over RXFP1. *J. Med. Chem.* 55 1671–1681. 10.1021/jm201505p 22257012

[B40] Smith C. M. Chua B. E. Zhang C. Walker A. W. Haidar M. Hawkes D. (2014). Central injection of relaxin-3 receptor (RXFP3) antagonist peptides reduces motivated food seeking and consumption in C57BL/6J mice. *Behav. Brain Res.* 268 117–126. 10.1016/j.bbr.2014.03.037 24681162

[B41] Smith C. M. Hosken I. T. Downer N. L. Chua B. E. Hossain M. A. Wade J. D. (2013). Pharmacological activation of RXFP3 is not orexigenic in C57BL/6J mice. *Ital. J. Anat. Embryol.* 118 (Suppl 1) 52–55. 24640572

[B42] Smith C. M. Shen P. J. Banerjee A. Bonaventure P. Ma S. Bathgate R. A. (2010). Distribution of relaxin-3 and RXFP3 within arousal, stress, affective, and cognitive circuits of mouse brain. *J. Comp. Neurol.* 518 4016–4045. 10.1002/cne.22442 20737598

[B43] Sohn J.-W. (2015). Network of hypothalamic neurons that control appetite. *BMB Rep.* 48 229–233. 10.5483/bmbrep.2015.48.4.272 25560696 PMC4436859

[B44] Volkow N. D. Wise R. A. Baler R. (2017). The dopamine motive system: implications for drug and food addiction. *Nat. Rev. Neurosci.* 18 741–752. 10.1038/nrn.2017.130 29142296

[B45] Yang X. W. Gong S. (2005). An overview on the generation of BAC transgenic mice for neuroscience research. *Curr. Protoc. Neurosci.* Chapter 5 Unit 5.20. 10.1002/0471142301.ns0520s31 18428622

[B46] Zhang C. Baimoukhametova D. V. Smith C. M. Bains J. S. Gundlach A. L. (2017). Relaxin-3/RXFP3 signalling in mouse hypothalamus: no effect of RXFP3 activation on corticosterone, despite reduced presynaptic excitatory input onto paraventricular CRH neurons in vitro. *Psychopharmacology* 234 1725–1739. 10.1007/s00213-017-4575-z 28314951

[B47] Zhang T. A. Placzek A. N. Dani J. A. (2010). In vitro identification and electrophysiological characterization of dopamine neurons in the ventral tegmental area. *Neuropharmacology* 59 431–436. 10.1016/j.neuropharm.2010.06.004 20600174 PMC2946471

[B48] Zhang X. Van Den Pol A. N. (2015). Dopamine/tyrosine hydroxylase neurons of the hypothalamic arcuate nucleus release GABA, communicate with dopaminergic and other arcuate neurons, and respond to dynorphin, met-enkephalin, and oxytocin. *J. Neurosci.* 35 14966–14982. 10.1523/jneurosci.0293-15.2015 26558770 PMC4642233

[B49] Zhang X. Van Den Pol A. N. (2016). Hypothalamic arcuate nucleus tyrosine hydroxylase neurons play orexigenic role in energy homeostasis. *Nat. Neurosci.* 19 1341–1347. 10.1038/nn.4372 27548245 PMC6402046

